# Transcriptomic Analysis of *Pseudomonas aeruginosa* Response to Pine Honey via RNA Sequencing Indicates Multiple Mechanisms of Antibacterial Activity

**DOI:** 10.3390/foods10050936

**Published:** 2021-04-24

**Authors:** Ioannis Kafantaris, Christina Tsadila, Marios Nikolaidis, Eleni Tsavea, Tilemachos G. Dimitriou, Ioannis Iliopoulos, Grigoris D. Amoutzias, Dimitris Mossialos

**Affiliations:** 1 Microbial Biotechnology-Molecular Bacteriology-Virology Laboratory, Department of Biochemistry & Biotechnology, University of Thessaly, Biopolis, 41500 Larissa, Greece; kafantarisioannis@gmail.com (I.K.); tsadila@uth.gr (C.T.); elenats89@hotmail.com (E.T.); tidimitr@bio.uth.gr (T.G.D.); 2 Bioinformatics Laboratory, Department of Biochemistry & Biotechnology, University of Thessaly, Biopolis, 41500 Larissa, Greece; marionik23@gmail.com (M.N.); amoutzias@bio.uth.gr (G.D.A.); 3 Department of Basic Sciences, School of Medicine, University of Crete, 71003 Heraklion, Greece; iliopj@med.uoc.gr

**Keywords:** *Pseudomonas aeruginosa*, pine honey, RNA-sequencing, antimicrobial activity, transcriptomics, biological process

## Abstract

Pine honey is a unique type of honeydew honey produced exclusively in Eastern Mediterranean countries like Greece and Turkey. Although the antioxidant and anti-inflammatory properties of pine honey are well documented, few studies have investigated so far its antibacterial activity. This study investigates the antibacterial effects of pine honey against *P*. *aeruginosa* PA14 at the molecular level using a global transcriptome approach via RNA-sequencing. Pine honey treatment was applied at sub-inhibitory concentration and short exposure time (0.5× of minimum inhibitory concentration –MIC- for 45 min). Pine honey induced the differential expression (>two-fold change and *p* ≤ 0.05) of 463 genes, with 274 of them being down-regulated and 189 being up-regulated. Gene ontology (GO) analysis revealed that pine honey affected a wide range of biological processes (BP). The most affected down-regulated BP GO terms were oxidation-reduction process, transmembrane transport, proteolysis, signal transduction, biosynthetic process, phenazine biosynthetic process, bacterial chemotaxis, and antibiotic biosynthetic process. The up-regulated BP terms, affected by pine honey treatment, were those related to the regulation of DNA-templated transcription, siderophore transport, and phosphorylation. Pathway analysis revealed that pine honey treatment significantly affected two-component regulatory systems, ABC transporter systems, quorum sensing, bacterial chemotaxis, biofilm formation and SOS response. These data collectively indicate that multiple mechanisms of action are implicated in antibacterial activity exerted by pine honey against *P*. *aeruginosa*.

## 1. Introduction

*Pseudomonas aeruginosa* is a ubiquitous, Gram-negative opportunistic human pathogen that can cause acute and chronic human infections in hospitalized or immune-compromised patients [1,2]. Typically, it infects the airway, urinary tract, burns, wounds, surgical site infections and also causes systemic blood infections that can lead to death [3]. The pathogenesis of *P*. *aeruginosa* is attributed to a variety of virulence factors, such as the cytotoxic pigment pyocyanin, the major siderophore pyoverdine, alkaline protease, elastase, exotoxins, flagella, and biofilm formation [4]. In addition, core genome analyses have revealed a distinct set of *P*. *aeruginosa* specific genes, related to its pathogenicity and lifestyle [5]. *P*. *aeruginosa* can adapt to a wide variety of environmental conditions and exhibits a remarkable high multidrug resistance by the formation of biofilms [6,7,8]. Considering its high prevalence associated with high mortality rates and lack of treatment options, this pathogen has been identified by the World Health Organization as a critical research priority for the development of alternative drugs and novel therapeutic strategies [9].

Recently, diverse natural products exerting antimicrobial activity have been widely investigated as alternative therapeutic agents to combat multidrug resistant pathogens. Honey, a natural product of honey bees, has been traditionally used in treating wounds and infectious diseases [10,11,12]. Many studies have proved the antimicrobial activity of different honey types against a plethora of pathogenic bacteria [13,14,15,16,17]. Previous studies conducted by our research group have also demonstrated that Greek and Cypriot honeys of diverse botanical origin exhibited potent antibacterial activity [18,19,20]. The antibacterial activity of honey to a wide range of pathogens is due to multiple factors including hydrogen peroxide (H_2_O_2_), low pH, methylglyoxal (MGO), antimicrobial peptides, and osmotic stress [16,21,22]. Several studies have shown numerous biological processes in bacteria that may be affected by honey such as protein synthesis, quorum sensing (QS), motility, biofilm formation, as well as response to oxidative stress [23,24,25,26].

Pine honey, is a unique type of honeydew honey produced in Eastern Mediterranean *Pinus brutia* and *Pinus halepensis* Miller forests, located in Greece and Turkey [27]. It is produced by bees which collect honeydew (sugary secretions) eliminated by the insect *Marchalina hellenica* (Gennadius), when feeding on certain pine trees [28]. Pine honey has an impressive pearl-amber color with characteristic metallic highlights, a spicy taste, as well as a thick texture. All the above characteristics combined with its natural property not to crystallize and its high content of minerals (potassium, calcium, iron, phosphorus, magnesium, sodium, and zinc), make pine honey a natural product of significant economic value [29]. It is estimated that in Greece and Turkey pine honey represents, 65% and 50% of the total annual honey production, respectively [30,31]. Although the antioxidant and anti-inflammatory properties of pine honey are well documented [32,33,34,35], few studies have investigated its antibacterial activity [18,36,37]. 

RNA sequencing (RNA-seq) is a cutting-edge technology for transcriptome profiling that can provide measurements of genome-wide quantitative analysis of all transcripts with high accuracy and sensitivity [38,39]. In addition, RNA-seq can reveal specific biological processes, affected in the presence of natural products or drugs [40,41]. To our knowledge, this is the first study that employs a global transcriptome approach via RNA-seq analysis to investigate the antibacterial effects of pine honey at the molecular level using as a model microorganism the *P*. *aeruginosa* PA14 strain.

## 2. Materials and Methods

### 2.1. Honey Samples

Pine honey was harvested in August 2019 from an apiary located in Chalkidiki area (Greece). After the collection, the sample was stored in glass container at room temperature in the dark. Manuka honey UMF 24+ (MGO 1122), Steens™, New Zealand (Batch No B084E3) was also used in this study.

### 2.2. Bacterial Strain, Growth Media, and Culture Conditions

The antibacterial activities of the pine and manuka honeys were tested upon *Pseudomonas aeruginosa* PA14 strain. The bacterial strain was routinely grown in Mueller-Hinton (MH) broth or MH agar (Lab M, Bury, UK) at 37 °C.

### 2.3. Assessment of the Minimum Inhibitory Concentration (MIC) and Minimum Bactericidal Concentration (MBC)

The minimum inhibitory concentration (MIC) of the tested honey sample was assessed in sterile 96-well polystyrene microtiter plate (Kisker Biotech GmbH & Co. KG, Steinfurt, Germany) using a spectrophotometric bioassay, as previously described [19]. Briefly, overnight bacterial culture grown in MH broth was adjusted to a 0.5 McFarland turbidity standard (~1.5 × 10^8^ cfu/mL). Approximately 5 × 10^4^ cfu in 10 μL MH broth was added to 190 μL of diluted test honey in MH broth. The control wells contained only MH broth, inoculated with bacteria. The optical density (OD) was determined at 630 nm using an EL x808 Absorbance microplate reader (BioTek Instruments, Inc., Winooski, VT, USA) before (t = 0) and after 24 h of incubation (t = 24) at 37 °C. The OD for each replicate well at t = 0 was subtracted from the OD of the same replicate well at t = 24. The growth inhibition at each honey dilution was measured using the formula: % inhibition = 1 − (OD test well/OD of corresponding control well) × 100. MIC was defined as the lowest honey concentration which results in 100% growth inhibition.

The MBC is the lowest concentration of any antibacterial agent that could kill tested bacteria. The MBC was determined by transferring a small quantity of sample contained in each replicate well of the microtiter plates to MH agar plates by using a microplate replicator (Boekel Scientific, Waltham, PA, USA). The plate was incubated at 37 °C for 24 h. The MBC was determined as the lowest honey concentration at which no grown colonies were observed [42].

### 2.4. Assessment of the Antibacterial Activity Attributed to Hydrogen Peroxide and Proteinaceous Compounds

The MIC of honey treated with bovine catalase or proteinase K was assessed in comparison to the untreated honey as previously described [18,43]. Briefly, 50% (*v*/*v*) honey in MH broth containing 100 mg/mL proteinase K (Blirt, Gdansk, Poland) or 600 U/mL bovine catalase (Serva, Heidelberg, Germany) was incubated for 16 h at 37 °C, then diluted and tested as described above. An increased MIC of treated honey compared to the untreated honey has shown that the antibacterial activity of tested honey was attributable to hydrogen peroxide and/or proteinaceous compounds, respectively.

### 2.5. Total RNA Isolation and RNA Sequencing

*Pseudomonas aeruginosa* PA14 culture was prepared in MH broth to an initial optical density at 600 nm (OD_600_) of 0.05 and then incubated in a 250-mL cell culture conical flask (Erlenmeyer, Duran) at 37 °C with shaking at 200 rpm until reaching mid-exponential phase (OD_600_ of 0.4). Cultures were then split into two conical sterile falcons (Falcon, Corning): one falcon contained 30 mL of untreated culture (control) and the second falcon (30 mL) contained the culture and the treatment at a final concentration of roughly 0.5× MIC of pine honey (4.5% *v*/*v*). Each culture was grown for an additional 45 min before total RNA isolation. The control and the treated culture were then split into three technical replicates (10 mL each). Total RNA from each replicate was isolated using a NucleoSpin RNA isolation kit (Macherey-Nagel) and DNA removed with DNase I, according to the manufacturer’s protocol. Samples were analyzed spectrophotometrically using a micro-volume UV-Vis instrument (Quawell, San Jose, CA, USA) for quantification and purity assessment. All RNA samples had an A_260_:A_280_ ratio between 1.8 and 2.0. RNA integrity was initially verified by 1% agarose gel electrophoresis. The six samples (3 controls and 3 treated) were shipped to Macrogen (Seoul, South Korea) for rRNA depletion using a NEBNext Bacterial rRNA removal kit (Illumina, San Diego, CA, USA), library preparation using the TruSeq stranded total RNA kit (Illumina, San Diego, CA, USA), and subsequent 150-bp paired-end RNA sequencing on a NovaSeq6000 platform (Illumina, San Diego, CA, USA). RNA integrity was further evaluated using the RNA Nano 6000 Assay Kit of the Agilent Bioanalyzer 2100 system (Agilent Technologies, Santa Clara, CA, USA); all samples demonstrated RNA integrity number (RIN) > 8.0.

### 2.6. Bioinformatics Analysis of the Differentially Expressed Genes (DEGs)

The fastq files were downloaded from Macrogen, with adapter trimming applied (TruSeq3 paired-ended) and their read quality was initially assessed using FASTQC (version 0.11.5) (http://www.bioinformatics.babraham.ac.uk/projects/fastqc, accessed on 20 December 2020). Subsequently, the reads were trimmed with Trimmomatic (version 0.38-default parameters) [44] and their quality was again assessed. Reads were aligned to the *P*. *aeruginosa* PA14 genome (NCBI reference sequence, NC_008463.1; GenBank accession number CP000438.1; assembly GCA_000014625.1) using the alignment program HISAT2 [45] and subsequently the number of reads that mapped to each gene was counted using the feature counts tool (version 1.6.4) with default parameters [46]. After mapping and counting, differential expression analysis, between control and treated samples, was carried out using the DESeq2 package (version 2.11.39) [47]. Gene annotation was carried out using the tool of DESeq2 package and the appropriate file (gtf) from the assembly (GCA_000014625.1). Genes whose expression displayed an average fold change >2 and was statistically significant (adjusted *p* value ≤ 0.05) were considered differentially expressed (DEGs). In order to understand more profoundly the biological functions and the metabolic pathways of the identified genes, the DEGs were functionally classified due to Gene Ontology (GO), using the Goseq tool (version 3.12) [48] and Kyoto Encyclopedia of Genes and Genomes (KEGG) database (http://www.genome.jp/kegg/, accessed on 27 December 2020) [49]. Go annotation and KEGG classifications were downloaded from the Pseudomonas Community Annotation Project (PseudoCAP) [50]. 

A second RNA sequencing analysis using a different pipeline was also conducted for assessing the robustness of the RNA-seq analysis conclusions. Briefly, the fastq files were trimmed with minimum contig length parameters and the quality of the final reads was inspected with FastQC. The trimmed fastq files were used for the de novo assembly of the *P*. *aeruginosa* PA14 transcriptome with the Trinity software (default parameters) [51], using all samples together. The resulting 5674 Trinity contigs were filtered to keep the longest isoform of each trinity gene, thus retaining 4903 contigs. The 4903 contigs were fed to TransDecoder software (https://github.com/TransDecoder/TransDecoder, accessed on 15 January 2021) with default parameters to identify the putative ORFs. Next the resulting 15,549 TransDecoder CDS were used as database for BLASTn search while the *P*. *aeruginosa* PA14 (GCA_000014625.1) CDS were used as a query with e-value cut-off 1 × 10^−5^. The best BLAST hit was kept for each *P*. *aeruginosa* PA14 gene, resulting in 4614 TransDecoder CDS. Next, Bowtie2 [52] within Trinity was used to align the reads back to the 4,614 TransDecoder CDS. In addition to the Trinity de novo assembly, a reference guided analysis was also performed by aligning the trimmed reads to the *P*. *aeruginosa* PA14 CDS with Bowtie2. Identification of DEGs in both analyses was conducted with the edgeR package [53] within Trinity, using default parameters.

## 3. Results and Discussion

### 3.1. Antibacterial Activity of Pine Honey against P. aeruginosa PA14

In order to investigate the activity of pine honey against *P*. *aeruginosa*, MIC and MBC values were determined. Data are presented in Table 1. 

The results clearly demonstrate that pine honey and manuka exert high anti-bacterial activity since both inhibited *P*. *aeruginosa* at 9% (*v*/*v*). Furthermore, pine honey was bactericidal at 9% (*v*/*v*), while manuka was bactericidal at 11% (*v*/*v*). In order to investigate the mechanisms which may contribute to the anti-bacterial activity, pine honey was treated with catalase and proteinase K. The proteinase K treated pine honey exhibited MIC value 9% (*v*/*v*) against *P*. *aeruginosa*, which is the same as the untreated honey, while the catalase treated honey exhibited higher MIC value 20% (*v*/*v*) indicating that the anti-bacterial activity of the pine honey was mainly attributable to hydrogen peroxide and not to proteinaceous compounds (Table 1). On the other hand, it is known that MGO is the main antimicrobial compound in manuka honey [22].

### 3.2. Effects of Pine Honey on the Transcriptomic Profile of P. aeruginosa PA14

#### 3.2.1. Global Response of *P. aeruginosa* to Pine Honey Treatment

The molecular response of *P*. *aeruginosa* to pine honey was investigated using RNA-Seq. Pine honey treatment was applied at sub-inhibitory concentration and short exposure time (0.5× MIC for 45 min), since this approach induces more specific response and reduces indirect effects [54]. A sub-inhibitory concentration may act as stress inducer or cues/coercion on receiver bacteria [55].

**Figure 1:**
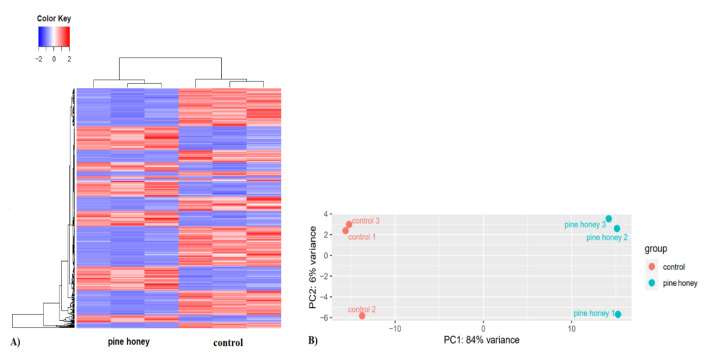
Transcriptional response of *P*. *aeruginosa* PA14 treated at mid-exponential phase with pine honey for 45 min at 0.5× MIC. (**A**) Clustered heatmap (based on Euclidean measures and complete agglomeration) of all DEGs (>two fold changes and *p* value ≤ 0.05) in *P*. *aeruginosa* across pine honey treatment and control. Each column represents one sample, and each row represents one gene. The red and blue gradients indicate up- and down-regulated gene expression, respectively. (**B**) Bi-plot of the principal-component analysis of DESeq2 normalized read counts (all coding genes) for pine honey treatment (green) and the control (red), split into technical replicates.

**Figure 2:**
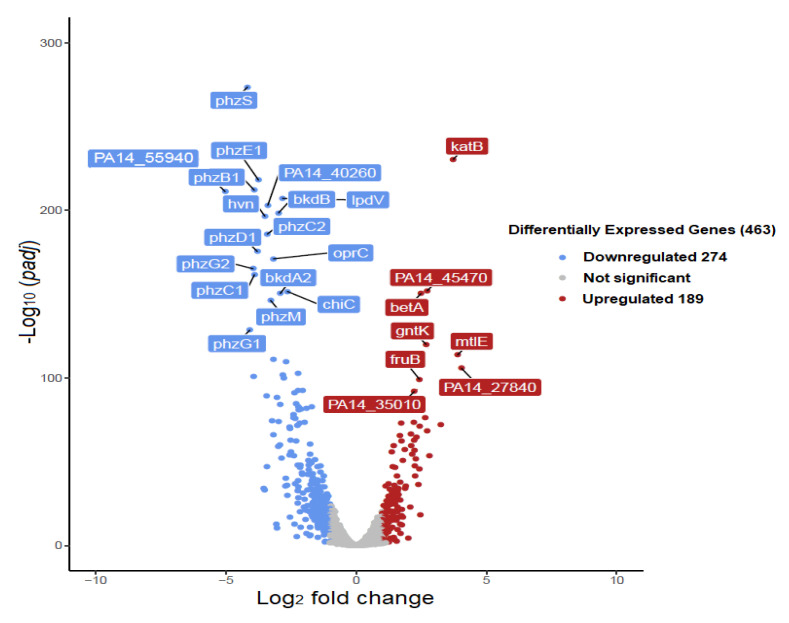
Volcano plot of differentially expressed genes (DEGs) based on RNA-seq analysis of untreated and pine honey-treated *Pseudomonas aeruginosa* PA14. Each gene is represented by a dot in the graph and the most differentially expressed up-and down-regulated genes are labeled in each plot. The x-axis and y-axis represent the log_2_ value of the fold change and the t-statistic as -log_10_ of the *p*-value, respectively. The genes represented in red (up-regulated) and blue (down-regulated) are differentially expressed genes with >two fold changes and a *p* value ≤ 0.05, while gray dots show genes with no significant difference compared to the control.

Data analysis of DEGs was conducted using three different pipelines, leading to very similar results and conclusions. RNA-seq analysis, using the pipeline (HISAT2-featurecounts-DESeq2), revealed that pine honey significantly affects the transcriptomic profile of *P*. *aeruginosa* PA14 compared to the control, with changes to the expression of 2543 out of 5964 coding sequences (42.6%; *p* ≤ 0.05). Of those 2543 genes, 1257 were up-regulated (21% of all coding genes) and 1286 were down-regulated (21.6%). The second pipeline (de novo Trinity-edgeR) revealed that pine honey induced the differential expression of 2115 out of 4673 genes (45.2%; *p* ≤ 0.05), where 1112 were up-regulated (23.8% of all coding genes) and 1103 were down-regulated (23.6%). In addition, the third pipeline (reference genome guided analysis-Bowtie2-edgeR) showed that pine honey induced the differential expression of 2451 out of 5964 coding sequences (41.1%; *p* ≤ 0.05) where 1195 were up-regulated (20% of all coding genes) and 1256 were down-regulated (21.1%). In a similar study, treatment of *P*. *aeruginosa* PA14 with manuka honey induced the differential expression of 3177 genes (54%; *p* ≤ 0.05) with 1646 of them being up-regulated (representing 28% of all coding genes) and 1531 being down-regulated (26% of all coding genes) [56]. Genome-wide expression changes were visualized as heatmap and volcano plot to identify specific genes with high fold changes and statistical significance. Results of hierarchical clustering and volcano plot are shown in Figure 1A and Figure 2. 

The results using the first pipeline (HISAT2-featurecounts-DESeq2) showed that pine honey treatment strongly induced the differential expression (log_2_FC > 1, meaning >two-fold change and *p* ≤ 0.05) of 463 genes (7.8% of all coding sequences) including 274 down-regulated and 189 up-regulated genes (Table S1). The results of the second pipeline (de novo Trinity-edgeR), revealed that pine honey treatment induced the differential expression (log_2_FC > 1 and *p* ≤ 0.05) of 440 genes (9.4% of all coding sequences) including 265 down-regulated and 175 up-regulated genes (Table S2). In addition, the last pipeline (reference guided analysis-Bowtie2-edgeR) showed that pine honey induced the differential expression (log_2_FC > 1 and *p* ≤ 0.05) of 482 genes (8.1% of all coding sequences) including 192 up-regulated and 290 down-regulated genes (Table S3). Further data analysis regarding DEGs was conducted using the pipeline (HISAT2-featurecounts-DESeq2). Compared to the study conducted by Bouzo et al. [56], treatment of *P*. *aeruginosa* PA14 with manuka honey highly induced the differential expression of 235 genes (log_2_FC > 2 meaning >four-fold change and *p* ≤ 0.05) including more up-regulated than down-regulated genes. In Figure 2, genes that were significantly differentially expressed are presented in red (up-regulated) and blue color (down-regulated). The most up- and down-regulated genes are labeled in each plot. In addition, Figure 1B shows the bi-plot of the principal-component analysis of DESeq2 normalized read counts (all coding genes) for pine honey treatment (green) and the control (red), split into technical replicates. Principal component analysis (PCA) confirmed that the effect of pine honey on *P*. *aeruginosa* differed significantly relative to the control (Figure 1B).

#### 3.2.2. Top Up- and Down-Regulated DEGs

In the pine honey-treated samples, the genes *katB*, PA14_45470, *betA*, *gntK*, *mtlE*, *fruB*, PA14_27840, and PA14_35010 were among the top up-regulated. These genes encode the catalase enzyme *katB* (log_2_FC 3.72), a putative glutathione S-transferase, the choline dehydrogenase *betA*, a gluconokinase, a putative binding protein component of ABC maltose/mannitol transporter (log_2_FC 3.89), a putative phosphotransferase system fructose-specific component, a putative copper-binding protein (log_2_FC 4.01) and a hypothetical protein respectively (Figure 2). It is documented that the catalase enzyme *katB* and glutathione S-transferases are induced in the presence of hydrogen peroxide. Moreover, these enzymes play multiple crucial roles in oxidative stress protection and bacterial virulence in *P*. *aeruginosa* [57,58,59]. Another enzyme, the choline dehydrogenase *betA* contributes toward the hyperosmotic stress resistance in *Pseudomonas protegens* [60]. Therefore the observed transcriptional response clearly shows that *P*. *aeruginosa* cells attempt to adapt to the hostile environment of pine honey, which is characterized by the presence of hydrogen peroxide and high osmolarity.

Among the genes that were strongly down-regulated were those encoding proteins involved in phenazine biosynthesis *phzB*1, *C*1, *C*2, *D*1, *E*1 (log_2_FC ranged from −3.40 to −3.90), PA14_55940 (log_2_FC −5.20) and PA14_40260 (log_2_FC −3.39) encoding a putative pilus assembly protein and a conserved hypothetical protein, respectively. Interestingly, KEEG pathway analysis (see also further below) revealed that PA14_40260 encodes a protein involved in the pathway of quorum sensing whereas, curated search in both KEEG and PseudoCAP databases revealed that PA14_55940, the most down-regulated gene in the presence of pine honey, encodes a bacterial motility protein (fimbriae associated protein Flp/Fap pilin component) of the protein secretion/export apparatus (Type II secretion system) [49,50]. Our observations are in accordance with a relevant study, where manuka honey reduced the motility of *P*. *aeruginosa* through the suppression of flagellin-associated genes [25]. It is plausible that pine honey reduces in a similar way the motility thus reducing *P*. *aeruginosa* virulence. 

Other genes that were also strongly down-regulated include *phzS* (log_2_FC −4.16) and M (log_2_FC −3.28) encoding a flavin-containing monooxygenase and a probable phenazine-specific methyltransferase respectively, *oprC* (log_2_FC −3.17) encoding an outer membrane copper receptor (pores ion channels), *hvn* (log_2_FC −3.50) encoding a putative halovibrin protein, *bkdB* encoding a lipoamide acyltransferase component of branched-chain alpha-keto acid dehydrogenase complex E2, *lpdV* (lipoamide dehydrogenase-Val) and *chiC* that encode a chitinase (Figure 2).

#### 3.2.3. Gene Ontology (GO) Enrichment Analysis

**Figure 3:**
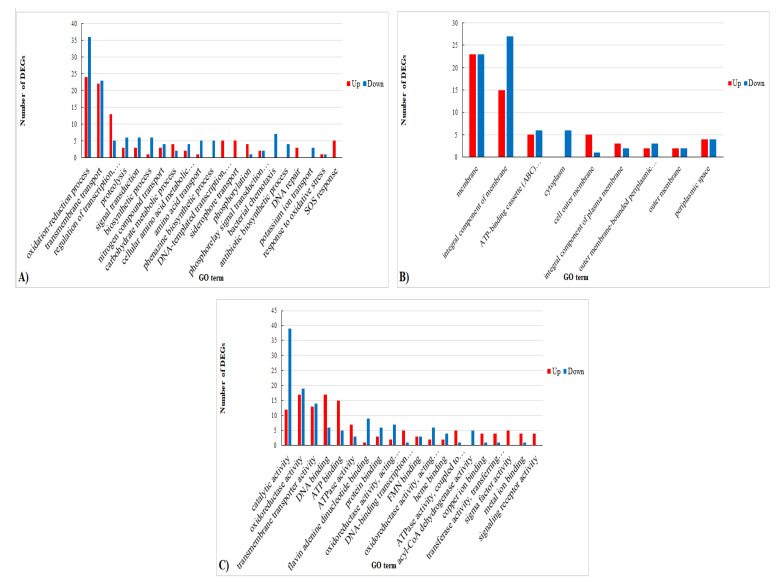
Gene Ontology categories of the DEGs. Three main categories were identified: (**A**) biological process (BP, total DEGs: 375, up-regulated: 177, down-regulated: 198) (**B**) cellular component (CC, total DEGs: 138, up-regulated: 62, down-regulated: 76) (**C**) molecular function (MF, total DEGs: 513, up-regulated: 233, down-regulated: 280).

In order to further investigate the biological functions and the metabolic pathways of DEGs in presence of pine honey, GO analysis was performed [50,61]. The most enriched GO categories among the DEGs are shown in Figure 3 and Supplementary Tables S4–S7. 

In the Biological Processes (BP) category (total DEGs: 375, up-regulated: 177, down-regulated: 198), the most enriched terms for up-regulated DEGs in presence of pine honey were related to “regulation of DNA-templated transcription,” “siderophore transport,” and “phosphorylation” whereas, in contrast, the most enriched BP GO terms for down-regulated DEGs were “oxidation-reduction process,” “transmembrane transport,” “proteolysis,” “signal transduction,” “biosynthetic process,” “phenazine biosynthetic process,” “bacterial chemotaxis,” and “antibiotic biosynthetic process” (Figure 3A, Table S5). In the Cellular Component (CC) category (total DEGs: 138, up-regulated: 62, down-regulated: 76), the most enriched terms for up-regulated DEGs in presence of pine honey were related to “cell outer membrane” and “integral component of plasma membrane” whereas the most enriched CC GO terms for down-regulated DEGs were “integral component of membrane,” “ATP-binding cassette (ABC) transporter complex,” and “cytoplasm” (Figure 3B, Table S6). In addition, in this category, the most enriched GO term was “membrane.” Furthermore, in the Molecular Function (MF) category (total DEGs: 513, up-regulated: 233, down-regulated: 280) the most enriched terms for up-regulated DEGs in presence of pine honey were “DNA and ATP binding” whereas, “catalytic activity” and “flavin adenine dinucleotide binding” were the most enriched terms for down-regulated DEGs. Other highly enriched MF GO terms were “oxidoreductase activity” and “transmembrane transporter activity” (Figure 3C, Table S7).

#### 3.2.4. KEGG Pathway Enrichment Analysis

**Figure 4:**
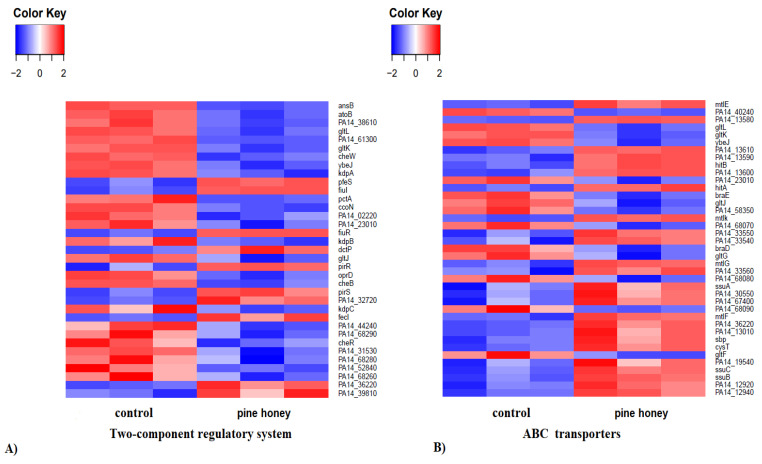
Heatmaps show log_2_FC data of DEGs implicated in: (**A**) two-component regulatory system. (**B**) ABC transporters, according to KEGG analysis. The red and blue gradients indicate up- and down-regulated gene expression.

Kyoto Encyclopedia of Genes and Genomes (KEGG) pathway enrichment analysis revealed that pine honey significantly affected several cellular pathways and induced the differential expression of genes involved in (but not limited to) two-component regulatory systems, ABC transporters, quorum sensing (QS), bacterial chemotaxis, and biofilm formation. Regarding the two-component regulatory systems, pine honey treatment caused significant up-regulation of 10 genes and down-regulation of 25 genes (Figure 4A). 

In treated samples, two genes (*pfeS* and *pirR*) encoding a sensor and response regulator respectively, were among the most up-regulated (log_2_FC 1.61 and 1.58, respectively). In contrast, the most down-regulated genes were *atoB*, PA14_38610, *ansB*, PA14_31530, *kdpA*, *B* and *C* (log_2_FC ranged from −1.62 to −3.22). These genes encode an acetyl-CoA acetyltransferase, a putative short-chain fatty acid transporter, a glutaminase-asparaginase, a putative acyl-CoA thiolase, and potassium-transporting ATPase chain ABC, respectively. Other down-regulated DEGs include *oprD* encoding an outer membrane porin and *cheW*, encoding a putative purine-binding chemotaxis protein. In the ABC transporter gene group the up-regulated were more prevalent than the down-regulated genes (Figure 4B). The most up-regulated genes were *mtlE*, *K*, *G* (log_2_FC ranged from 1.56 to 3.89) encoding putative ATP-binding component of ABC maltose/mannitol transporters whereas, the most down-regulated genes were PA14_40240 and *gltK*, *L* encoding a putative ATP-binding/permease fusion and, putative permease and ATP-binding component of ABC transporter system respectively. Interestingly, apart from the down-regulated genes *oprC* and *D*, encoding outer membrane porins, *oprB* (log_2_FC −1.65) encoding a glucose/carbohydrate outer membrane porin, and PA14_58410 (log_2_FC −1.55) encoding putative membrane porin, were also down-regulated. In contrast, the genes *mexF* and *E*, encoding putative RND efflux transporter and RND efflux membrane fusion protein precursor, were up-regulated (log_2_FC, 2.25 and 2.42, respectively). *OprC* is a porin abundant in the outer membrane vesicles involved in channel-forming and copper binding [62]. *OprC* transports copper, an essential trace element implicated in several physiological processes, into bacteria during copper deficiency. In a very recent study the authors showed that *oprC* deletion inhibited bacterial motility and quorum-sensing systems, as well as decreased lipopolysaccharide and pyocyanin levels in *P*. *aeruginosa* [62]. Interestingly, a previous study has shown that manuka honey decreased pyocyanin production in *P*. *aeruginosa* PA14, presumably via interaction with the MvfR quorum sensing network [63]. The *oprD* porin facilitates the diffusion of basic amino acids and peptides containing these residues. Moreover, it is implicated in carbapenem resistance [64]. On the other hand, the *oprB* porin has been associated with the diffusion of glucose across the outer membrane of *P*. *aeruginosa* thanks to the ABC transporter *glt* [65,66]. Raneri et al. [67] have demonstrated that *P*. *aeruginosa* mutants defective in glucose uptake have pleiotropic phenotype and attenuated virulence in non-mammal infection models. In this study, both *oprB* porin and *glt* ABC transporter were down-regulated. Previous studies have shown that reduced permeability of the outer membrane through *oprD* impairment and overexpression of the major resistance-nodulation-division (RND) efflux pump systems (MexAB-OprM, MexCD-OprJ, MexEF-OprN, and MexXY-OprM), contribute to carbapenem resistance in *P*. *aeruginosa* [68,69]. In this study, *oprD* is down-regulated in contrast to *mexF* and *mexE* (components of MexEF-OprN RND efflux pump system) which are up-regulated in the presence of pine honey. It is tempting to speculate that such differential gene expression might counteract the anti-bacterial activity of compounds (e.g., phytochemicals) contained in pine honey.

Furthermore, RNA-seq analysis revealed that a group of genes implicated in iron uptake and transport are up-regulated when *P*. *aeruginosa* PA14 is exposed to pine honey. These genes include *fptA*, *fecA*, *fpvA*, *piuA,* and *tonB* (log_2_FC ranged from 1.05 to 2.43) encoding the Fe(III)-pyochelin outer membrane receptor, a TonB-dependent siderophore receptor, the ferripyoverdine receptor, a putative outer membrane ferric siderophore receptor, and periplasmic protein TonB, respectively. Moreover, two genes *pchD* and *pchE*, implicated in pyochelin biosynthesis, were up-regulated (log_2_FC 1.13 and 1.14, respectively). Iron is a key nutrient, involved in many crucial biological processes. Therefore, it is essential for bacterial growth and virulence. In order to overcome restricted iron bioavailability, *P*. *aeruginosa* developed various strategies to acquire iron through the direct production of siderophores such as pyoverdine as well as pyochelin and the uptake of siderophores via TonB-dependent receptors (TBDRs) [70]. Several studies have shown that TBDRs could be employed in a “Trojan horse” strategy, in which the interaction between a siderophore and an antibiotic could significantly increase the antibiotic bioactivity, by facilitating its transport into the bacterial cell [71,72,73]. Previous reports have demonstrated the involvement of different TBDRs such as *piuA*, *fpvA*, *fecA,* and *fptA* in the uptake of siderophore-drug conjugates in *P*. *aeruginosa* [73,74]. Our data suggest that honey might impose an iron-limited environment for *P*. *aeruginosa*, which could be potentially exploited in combination with siderophore-antibiotic conjugates as an alternative approach to combat this multi-drug resistant pathogen.

**Figure 5:**
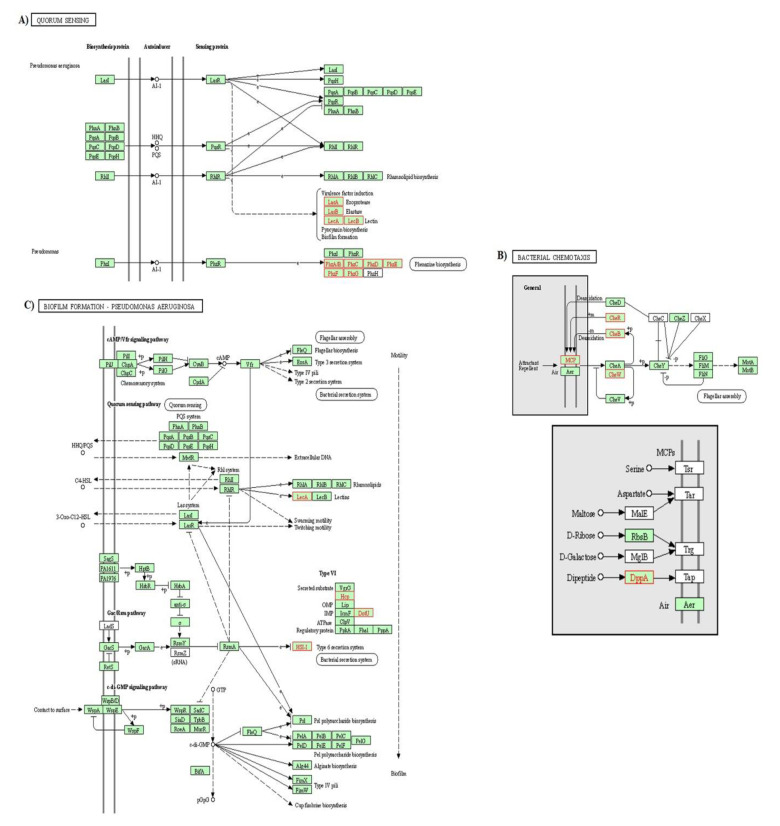
The KEGG pathways based on RNA high-throughput sequencing analysis of (**A**) quorum sensing, (**B**) bacterial chemotaxis, (**C**) biofilm formation. The red box indicate that the RNA expression of the gene is down-regulated, while the black box shows no changes in gene RNA expression.

Pine honey treatment significantly affected the expression of several genes involved in quorum sensing (QS), bacterial chemotaxis, and biofilm formation pathways (Figure 5A–C). 

Interestingly, pine honey treatment provoked significant down-regulation of almost all genes involved in the above pathways. The values of log_2_FC ranged from −1.02 to −5.20. The genes *phzG1* and *G2* (log_2_FC −4.08 and −3.95, respectively) encode a probable pyrodoxamine 5′-phosphate oxidase whereas, the genes *lasA*, *B,* and *lecB* (log_2_FC −1.90, −2.4, and −2.97 respectively) encode a staphylolytic exoprotease preproenzyme, an elastase, and a fucose-binding lectin PA-IIL, respectively. In the biofilm formation pathway the identified genes were *pa1L*, PA14_34050, PA14_34070, PA14_34100, PA14_34030, and PA14_34000 (log_2_FC ranged from −1.02 to −2.9) encoding a PA-I galactophilic lectin and conserved hypothetical proteins, respectively whereas, in the bacterial chemotaxis pathway the involved genes were PA14_61300, *cheW*, *pctA*, PA14_02220, PA14_58350, *cheB*, *cheR* (log_2_FC ranged from −1.02 to −1.67) encoding various chemotaxis proteins (i.e., methyltransferase, methyl-accepting and purine-binding). 

Furthermore, pine honey induced the differential expression of genes involved in SOS response such as *lexA*, *recA*, *N*, *X,* and PA14_25150 (log_2_FC ranged from 1.2 to 1.72). Similarly, Bouzo et al. (2020) have demonstrated that manuka honey significantly up-regulated a wide range of genes involved in SOS response.

Based on KEGG pathway and GO enrichment analysis, pine honey affected, at the transcriptome level, a wide range of biological processes and pathways in *P*. *aeruginosa*. The two-component regulatory system, the ABC transporter, and QS pathway were the most affected KEGG pathways in *P*. *aeruginosa*, since several up and down-regulated DEGs exhibited high fold changes (Figure 4 and Figure 5). A two-component regulatory system plays a substantial role in the pathogenicity, bacterial adaptation, and biofilm formation [75,76]. The two-component regulatory system KEGG pathway (also called “two-component signal transduction system”) enables bacteria to sense and respond to environmental or intracellular changes [77,78]. In this study, pine honey treatment induced the differential expression of several genes implicated in this pathway (Figure 4A). Among the down-regulated DEGs in the above pathway, *cheW*, *B* and *R*, PA14_02220 and *pctA* genes encode chemotaxis proteins and transducers, respectively. The *cheW*, *B* and *R* DEGs were also detected in the bacterial chemotaxis pathway (Figure 5B). Bacterial chemotaxis is the movement of bacterial cells in response to chemical stimuli [79]. According to Turner et al. [80], *cheW*, *B,* and *R* genes, are required in acute but not chronic wound infections. These data suggest that pine honey treatment might impair the two-component system and bacterial chemotaxis pathways thus reducing the ability of *P*. *aeruginosa* to sense environmental stimuli and adapt accordingly. In comparison to the study of Bouzo et al. [56], manuka honey treatment did not affect at the same extent the two-component regulatory system and bacterial chemotaxis pathways. 

Regarding the ABC transporter pathway, pine honey treatment caused significant up-regulation of 25 genes and down-regulation of 14 genes (Figure 4B). ABC (ATP-binding cassette) transporters play an important role in nutrients uptake [81]. In addition, ABC transporter and two-component regulatory systems have a pivotal role in antimicrobial drug resistance [82]. It might be that up-regulation of several ABC transporter genes might be related to nutrient uptake directly from pine honey (e.g., sugars).

Furthermore, KEEG analysis revealed that pine honey treatment significantly inhibited QS, bacterial chemotaxis, and biofilm formation pathways, since several key genes were down-regulated (Figure 5). In *P*. *aeruginosa*, three systems *las*, *rhl,* as well as *pqs*, which are forming an hierarchical network, play a crucial role in QS [83,84]. The *las* system positively regulates itself as well as the other two systems, while the *rhl* and *pqs* systems regulate each other (Figure 5A). In the first system, *lasI* catalyzes the synthesis of the signal molecule (AI-1), by binding *lasR* and activating the expression of many genes (*pqsA*, *B*, C, *D*, *E*, *H*, *R*, *phnA*, *B* and *rhlI*, *R*) [85,86]. In the *pqs* system [87], genes such as *pqsA*, *B*, *C*, *D*, *H,* and *phnA*, *B*, catalyze the synthesis of the signal molecules (HHQ or PQS), by binding *pqsR* and activating the expression of various genes, including *pqsR* as well as *rhlI*, *R*, whereas in the third system, *rhlI* catalyzes the synthesis of the signal molecule (AI-1), by binding *rhlR* and activating the expression of other target genes (*pqsR*, *phnA*, *B*, *rhlI*, *R* and *rhlA*, *B*) involved in the rhamnolipid biosynthesis [88]. Furthermore, in Figure 5A it is shown that the virulence factors *lasA* (exoprotease), *lasB* (elastase) and *lecB* (lectin), pyocyanin biosynthesis, and biofilm formation are co-regulated by the three QS systems (*las*, *pqs*, and *rhl*). In this study, pine honey treatment inhibited the expression of virulence genes such as *lasA*, *lasB*, *pa1L* (*lecA*) and *lecB* (Figure 5A). The gene *lecA* is also involved in the biofilm formation pathway. In addition, the genes of the operon *phzABCDEFG* involved in the phenazine biosynthesis, and the genes *phzS* and *M* implicated in the pyocyanin biosynthesis, were down-regulated in a similar manner (log_2_FC > 3). Previous studies have demonstrated that several enzymes of the biosynthetic operon *phzABCDEFG*, which is conserved across the fluorescent Pseudomonads, are involved in phenazine biosynthesis, through the conversion of chorismic acid to phenazine-1-carboxylic acid (PCA) [89,90]. *P*. *aeruginosa* has two functional copies (*phz1* and *phz2*) of this operon, which produce PCA. The conversion of PCA to phenazine-1-carboxamide as well as to 1-hydroxyphenazine is mediated by two genes *phzH* and *phzS*, respectively. A third additional gene *phzM* is involved in PCA conversion to 5-methylphenazine-1-carboxylic acid betaine, which is further converted to pyocyanin by the action of *phzS* [89,90,91]. A recent study showed that phenazine production is associated with the antibiotic tolerance in *P*. *aeruginosa* biofilms [92].

Regarding the biofilm formation pathway, KEEG analysis revealed that pine honey treatment down-regulated key genes including *pa1L* (lecA) that encode a PA-I galactophilic lectin. Additionally, several genes encoding conserved hypothetical proteins (HIS-I; PA14_34000, PA14_34030, PA14_34050, PA14_34070 and PA14_34100) were also inhibited (Figure 5C). Interestingly, pine honey treatment also down-regulated PA14_34030 (Hcp) and PA14_34110 (DotU) implicated in the type VI secretion system of *P*. *aeruginosa* (Figure 5C) and PA14_55940 (putative pilus assembly protein) gene of the protein secretion/export apparatus (Type II secretion system) (Figure 5C). In the bacterial chemotaxis pathway, besides *cheW*, *B* and *R* genes, pine honey also down-regulated PA14_58350 (DppA), PA14_61300, and PA14_02220 (MCP) (Figure 5B). Collectively, these results indicate that pine honey down-regulated several genes involved in the QS system (virulence factors, phenazine production, chemotaxis, and biofilm formation pathway) thus reducing the fitness of *P*. *aeruginosa* to initiate infection or biofilm formation.

In a very recent study, transcriptome analysis of *P. aeruginosa* biofilm treated with *Trigona* honey revealed that roughly 13.5% of the down-regulated genes were biofilm-associated genes. Additionally, in the pathways involved in biofilm formation, an ultimate decrease in the expression levels of the D-GMP signaling pathway and diguanylate cyclases genes implicated in c-di-GMP formation, has been observed [93].

In comparison to the study of Bouzo et al. [56], manuka honey mainly down-regulated genes of the three different QS systems (*las*, *rhl* and *pqs*) while in this study pine honey treatment demonstrated a direct inhibitory effect on genes encoding virulence factors and phenazine biosynthesis. Interestingly, pine honey also down-regulated genes implicated in bacterial chemotaxis, biofilm formation, and bacterial secretion pathways, indicating a broader mode of action on the QS system, while this does not occur at such extent following manuka honey treatment.

## 4. Conclusions

The present study is the first to employ a global transcriptomic approach, in order to investigate the antibacterial effects and mode of action of pine honey. RNA-seq analysis revealed that pine honey significantly affected the trascriptomic profile of *P*. *aeruginosa* by increasing significantly the expression of 189 genes and by reducing significantly the expression of 274 genes. Specifically, pine honey treatment exerted a broad range of action on several pathways and biological processes including oxidation-reduction process, transmembrane transport, proteolysis, regulation of DNA-templated transcription, two-component regulatory systems, ABC transporters, and SOS response. Interestingly, pine honey might inhibit quorum sensing, bacterial chemotaxis, and biofilm formation since several differentially expressed genes involved in the above pathways were strongly down-regulated. Overall, these data demonstrated that pine honey exerted an inhibitory effect in *P*. *aeruginosa* genome expression since more genes were down-regulated than up-regulated. These findings could potentially contribute to the treatment and control of *P*. *aeruginosa* infection and pathogenicity, helping to elucidate the molecular pathways and biological processes implicated in the antibacterial activity exerted by pine honey. Moreover, our results suggest that the use of pine honey in wound dressings could be an effective and economical approach to ameliorate wound healing.

## Figures and Tables

**Table 1:** Antibacterial activity of pine honey and manuka against *P*. *aeruginosa*.

Honey	MIC % (*v*/*v*) ^1^	MBC % (*v*/*v*) ^2^	MICp % (*v*/*v*) ^3^	MICc % (*v*/*v*) ^4^
pine honey	9	9	9	20
manuka	9	11	ND ^5^	ND

^1^ MIC, minimum inhibitory concentration. ^2^ MBC, minimum bactericidal concentration. ^3^ MICp, MIC values of proteinase K treated honey. ^4^ MICc, MIC values of catalase treated honey. ^5^ ND, not determined.

## Data Availability

The raw RNA-seq data are available at NCBI Sequence Read Archive (NCBI SRA) under BioProject accession no. PRJNA705535. https://www.ncbi.nlm.nih.gov/bioproject/?term=PRJNA705535 (accessed on 1 March 2021).

## Supplementary Materials

The following are available online at https://www.mdpi.com/article/10.3390/foods10050936/s1. Table S1: Differential gene expression results of HISAT2-DESeq2 package. Table S2: Differential gene expression results of de novo Trinity-edgeR package. Table S3: Differential gene expression results of reference genome guided analysis-Bowtie2-edgeR package. Table S4: Gene Ontology (all categories) of DEGs. Table S5: Gene Ontology (biological process category) of DEGs. Table S6: Gene Ontology (cellular component category) of DEGs. Table S7: Gene Ontology (molecular function category) of DEGs.
